# Synergistic Cytotoxicity of Cafestol and Kahweol With Doxorubicin in Breast Cancer Cells Involves TNF‑α Targeting and NF‑κB Downregulation

**DOI:** 10.1002/ardp.70359

**Published:** 2026-10-07

**Authors:** Layane D. Souza, Fábio Junior M. Novaes, Carlos Luan A. Passos, Ísis S. S. de Amorim, Pedro Nicolau‐Neto, Juliana A. Rodrigues, Adilson F. Teixeira, Christian Ferreira, Nathalia Alexia N. dos Santos, Priscila G. Camargo, Marcelle L. F. Bispo, Camilo Henrique S. Lima, Bruno Ricardo B. Pires, Adenilson de Sousa da Fonseca, Claudia M. Rezende, Eliane Fialho, Andre Luiz Mencalha

**Affiliations:** ^1^ Department of Biophysics and Biometrics, Roberto Alcantara Biology Institute State University of Rio de Janeiro Rio de Janeiro Brazil; ^2^ Chemistry Department Viçosa Federal University Viçosa Brazil; ^3^ Aromas Analysis Laboratory, Chemical Institute Federal University of Rio de Janeiro Rio de Janeiro Brazil; ^4^ Functional Foods Laboratory, Nutrition Institute Federal University of Rio de Janeiro Rio de Janeiro Brazil; ^5^ Molecular Carcinogenesis Program, National Cancer Institute Rio de Janeiro Brazil; ^6^ Laboratory of Synthesis of Medicinal Molecules, Chemistry Department State University of Londrina Londrina Brazil; ^7^ Chemistry Institute Federal University of Rio de Janeiro Rio de Janeiro Brazil

**Keywords:** breast cancer, coffee, diterpenes, NF‐κB, TNF‐α

## Abstract

Breast cancer (BC) is the most common cancer in women worldwide. Natural compounds have been studied to improve chemotherapy efficiency. Cafestol and kahweol (C&K) are diterpenes from coffee lipid fraction, with potential anticancer activity, whose effects and association with chemotherapeutic drug doxorubicin in BC cells are still unknown. Here, we evaluated the synergistic cytotoxic effects of the C&K with doxorubicin and their molecular mechanisms in BC cells. Cell viability was assessed by the MTT assay. Flow cytometry was used to analyze apoptosis, cell cycle, and NF‐κB levels. The microarray assay was performed to identify altered signaling pathways, and molecular docking was utilized to predict the interaction between C&K and TNF‐α. In addition, TCGA data were explored to evaluate the clinical association between the expression of C&K‐regulated genes and TNF‐α/NF‐κB pathway genes. C&K was cytotoxic and increased the doxorubicin cytotoxicity, augmented the sub‐G0/G1 phase, downregulated TNF‐α/NF‐κB pathway, decreased NF‐κB protein levels, and showed affinity for TNF‐α by molecular docking. Moreover, considering the C&K‐modulated genes, *BLOC1S1* acted as a protective factor for BC, and these genes were negatively correlated with TNF‐α/NF‐κB genes. Our data suggest that C&K have cytotoxic properties by inhibiting TNF‐α/NF‐κB pathway and interacting synergistically with doxorubicin in BC cells.

## Graphical abstract

Cafestol and kahweol (C&K) are cytotoxic to breast cancer cells and have a synergistic effect with doxorubicin. C&K interact with TNF‐α and downregulate the TNF‐α/NF‐κB pathway, underscoring their therapeutic potential for breast cancer. NIAID Visual & Medical Arts. Generic Cells. NIAID NIH BIOART Source. bioart.niaid.nih.gov/bioart/171. NIAID Visual & Medical Arts. Cell Death. NIAID NIH BIOART Source. bioart.niaid.nih.gov/bioart/71.

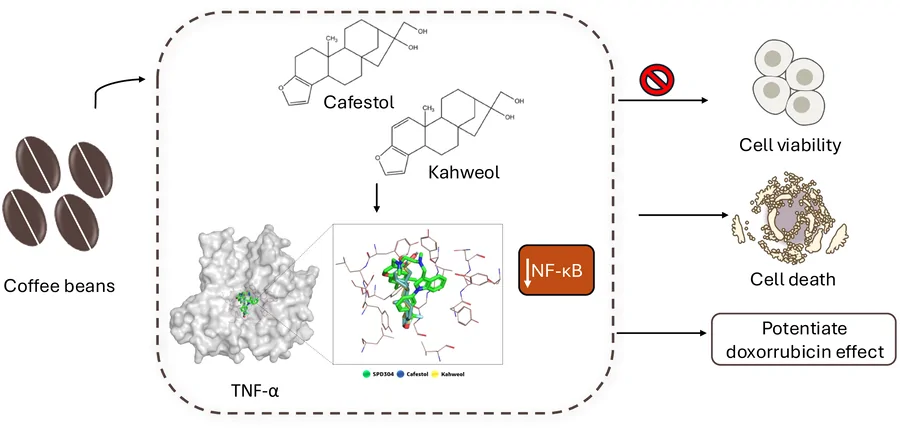

## 1 Introduction

Breast cancer (BC) is the most common cancer in women worldwide [1, 2]. Although the chemotherapy drug doxorubicin is widely used in the treatment of BC, new therapies are needed to reduce its adverse effects and overcome resistance [3]. In this sense, the association of natural compounds with conventional chemotherapeutics, such as doxorubicin, has shown synergistic effects and anticancer potential by improving anticancer effects through the reduction of doses and side effects and decreasing the probability of metastasis [4, 5].

In this context, cafestol and kahweol (C&K) are diterpenes obtained from coffee lipid fraction with anticancer potential [6, 7]. In addition, C&K demonstrated other bioactivities and potential pharmacological properties such as anti‐inflammatory, antioxidant, antiangiogenic, antidiabetic, antiosteoclastogenic, and hepatoprotective, which indicate its potential for medicine [8].

Studies have shown that these compounds are individually effective against cancer cell proliferation. Kahweol inhibits cell growth of several human cancer cell types, including leukemia, hepatocellular carcinoma, and breast cancer cells [9]. Cafestol induces apoptosis in breast cancer and glioblastoma cells [10], reduces cellular viability, and sensitizes head and neck squamous cell carcinoma cells to radiation [11]. Both also decrease the proliferation and migration of renal cancer cells [12].

Although the cytotoxic effects of cafestol and kaweol alone in cancer cells have been investigated, the effects of the C&K mixture and the combination of C&K and doxorubicin in breast cancer cells still need to be evidenced. Therefore, this study aimed to evaluate the cytotoxic and molecular effects in response to C&K and the combination of C&K with doxorubicin in breast cancer cells.

## 2 Results

### 2.1 Treatment With Cafestol and Kahweol (C&K) Is Cytotoxic to Breast Cancer Cells

To evaluate the cytotoxic effect of C&K, the MCF‐7 and MDA‐MB‐231 breast cancer cells and the MCF‐10A non‐tumorigenic breast cell line were treated with varying concentrations of C&K (10, 25, 50, 75, and 100 μM) for 48 and 72 h, and cells viabilities were assessed by MTT assay. We observed a decrease in breast cancer cells' viability in a dose‐dependent manner (Figure 1). The IC_50_ values for C&K treatment were 22.1 μM ± 4.10 (48 h) and 18.1 μM ± 0.67 (72 h) in MCF‐7 cells; while IC_50_ values were 30.4 μM ± 3.80 (48 h) and 26.4 μM ± 1.62 (72 h) for MDA‐MB‐231 cells. Additionally, IC_50_ values were 33.1 μM ± 0.21 (48 h) and 30.3 μM ± 1.42 (72 h) in MCF‐10A cells (Supporting Information S2: Table 1).

**Figure 1:**
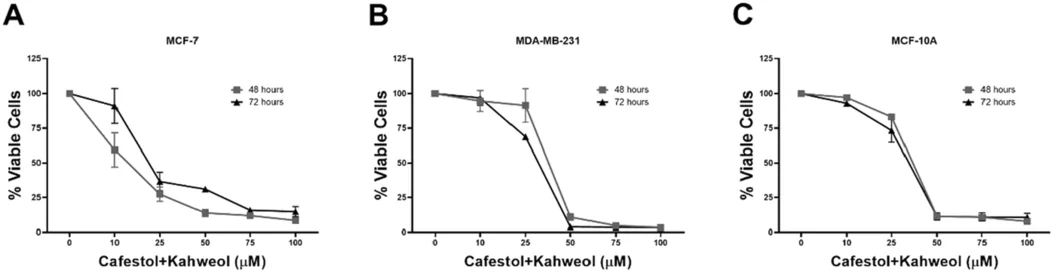
Evaluation of the cytotoxic effect of C&K on MCF‐7 and MDA‐MB‐231 breast cancer cells, and non‐tumorigenic MCF‐10A cells. Cell viability was analyzed via the MTT assay after treatment of MCF‐7 (A), MDA‐MB‐231 (B), and MCF‐10A (C) cells with C&K at concentrations ranging from 10 to 100 µM for 48 and 72 h. The results represent the means ± SEM from three experiments performed in triplicate.

The ratio between the IC_50_ of the non‐tumorigenic cells and the IC_50_ of the tumor cells represents the selectivity index (SI). The higher the ratio, the more selective a tested compound is on the tumor cell than on the non‐tumorigenic cell [13]. The SIs for C&K treatment in MCF‐7 cells were 1.50 and 1.67 at 48 and 72 h, respectively. For MDA‐MB‐231 cells, the SIs were 1.09 (48 h) and 1.15 (72 h) (Supporting Information S2: Table 1). Our findings demonstrate that C&K present the highest cytotoxic effects on breast cancer cells.

### 2.2 Co‐Treatment With C&K and Doxorubicin Promotes a Synergistic Cytotoxic Effect on MDA‐MB‐231 Cells

To analyze the synergistic effect of C&K with doxorubicin on the MDA‐MB‐231 cell line, since this cell line is more aggressive than MCF‐7 [14], we used the fractional inhibitory concentration index and the isobologram method. Thus, the IC_50_ values for both drugs were determined (Figure 2A–D, Supporting Information S2: Table 2). The calculated IC_50_ value for C&K was plotted on the *y* axis, and the IC_50_ values for doxorubicin were plotted on the *x* axis (Figure 2E). The two points were connected to form the line of additivity. The points on this line were classified as additive; points above the line indicate antagonistic effects, and points below the line indicate synergistic effects. The association of 17.52 μM C&K + 0.15 μM doxorubicin, 13.25 μM C&K + 0.31 μM doxorubicin, 9.98 μM C&K + 0.62 μM doxorubicin, and 4.89 μM C&K + 1.25 μM doxorubicin showed a synergistic effect, with FIC values of 0.63, 0.55, 0.56, and 0.66, respectively (Supporting Information S2: Table 2). The synergistic combination with a lower concentration of the chemotherapy drug doxorubicin was used in the following experiments. The data demonstrate that the effect of doxorubicin treatment can be improved when combined with the C&K, enabling the use of lower concentrations. It is worth highlighting that the combination of C&K with doxorubicin was cytotoxic and demonstrated synergism in the MDA‐MB‐231 aggressive triple‐negative cell line, which has not yet been demonstrated in the literature.

**Figure 2:**
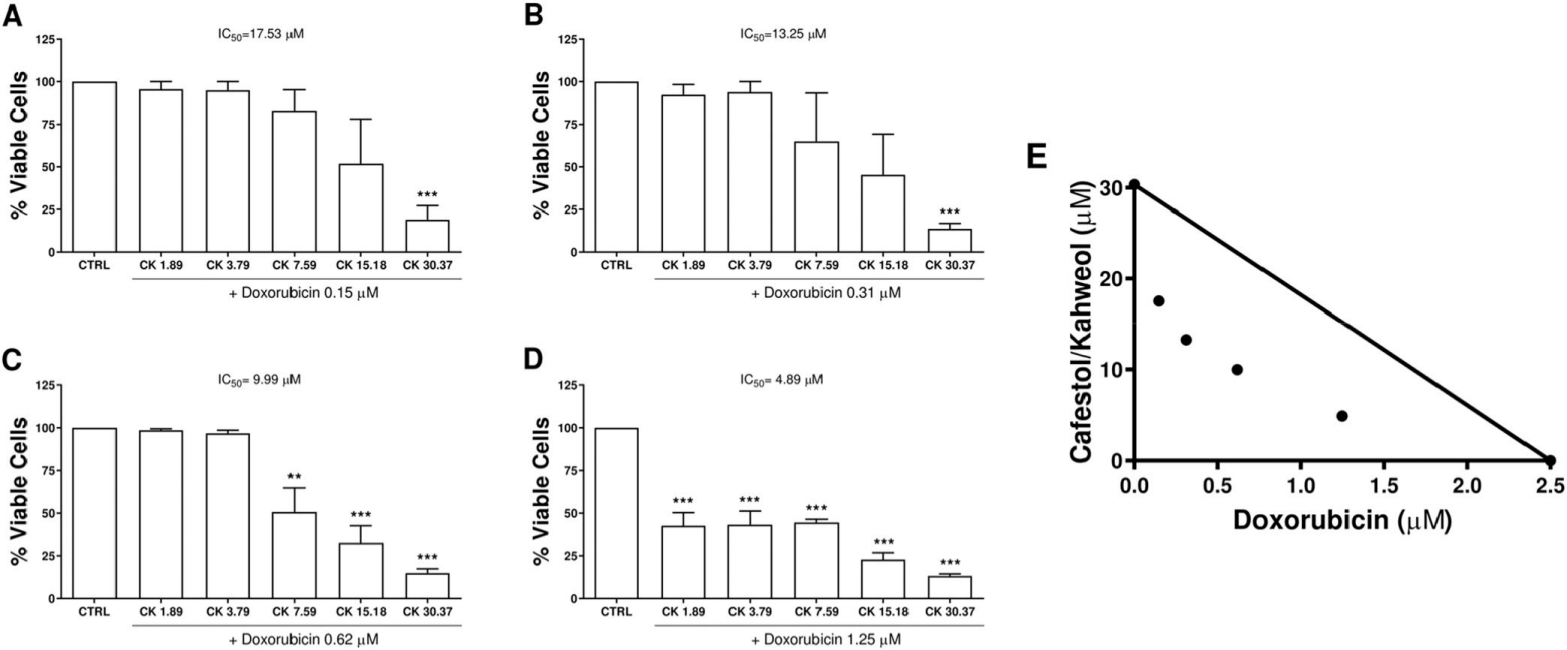
Analysis of the effect of the combination of C&K and doxorubicin on breast cancer cell viability. The cytotoxicity of co‐treatment of MDA‐MB‐231 cells with different concentrations of C&K and doxorubicin (0.15 µM (A), 0.31 µM (B), 0.62 µM, and 1.25 µM (D)) for 48 h was determined by the MTT assay. For the isobologram, cells were treated or not with the indicated concentration of C&K + DOX (E). The diagonal line of the isobologram is the line of additivity and represents all the additive theoretical combinations that should inhibit survival by 50%. The points below the additivity line represent synergistic combinations. The results represent the means ± SEM from three experiments performed in triplicate. **p < 0.001, ***p < 0.0001, in relation to control.

### 2.3 C&K Associated With Doxorubicin Promote Cell Death, Alter the Cell Cycle, and Increase ROS Levels

MDA‐MB‐231 cells were labeled with annexin‐V‐FITC and propidium iodide (PI) to determine the type of cell death in response to treatment with 30.40 μM C&K, 2.50 μM doxorubicin, and association of 17.52 μM C&K with 0.15 μM doxorubicin (Figure 3A–H). Our results demonstrate that C&K with doxorubicin increased the percentage of annexin‐V‐positive cells by 4.05‐fold, indicating cell death by apoptosis (Figure 3F). C&K, doxorubicin, and C&K with doxorubicin increased by 1.51‐, 1.31‐, and 1.32‐fold the percentage of PI‐positive cells, indicating cell death by necrosis (Figure 3G).

**Figure 3:**
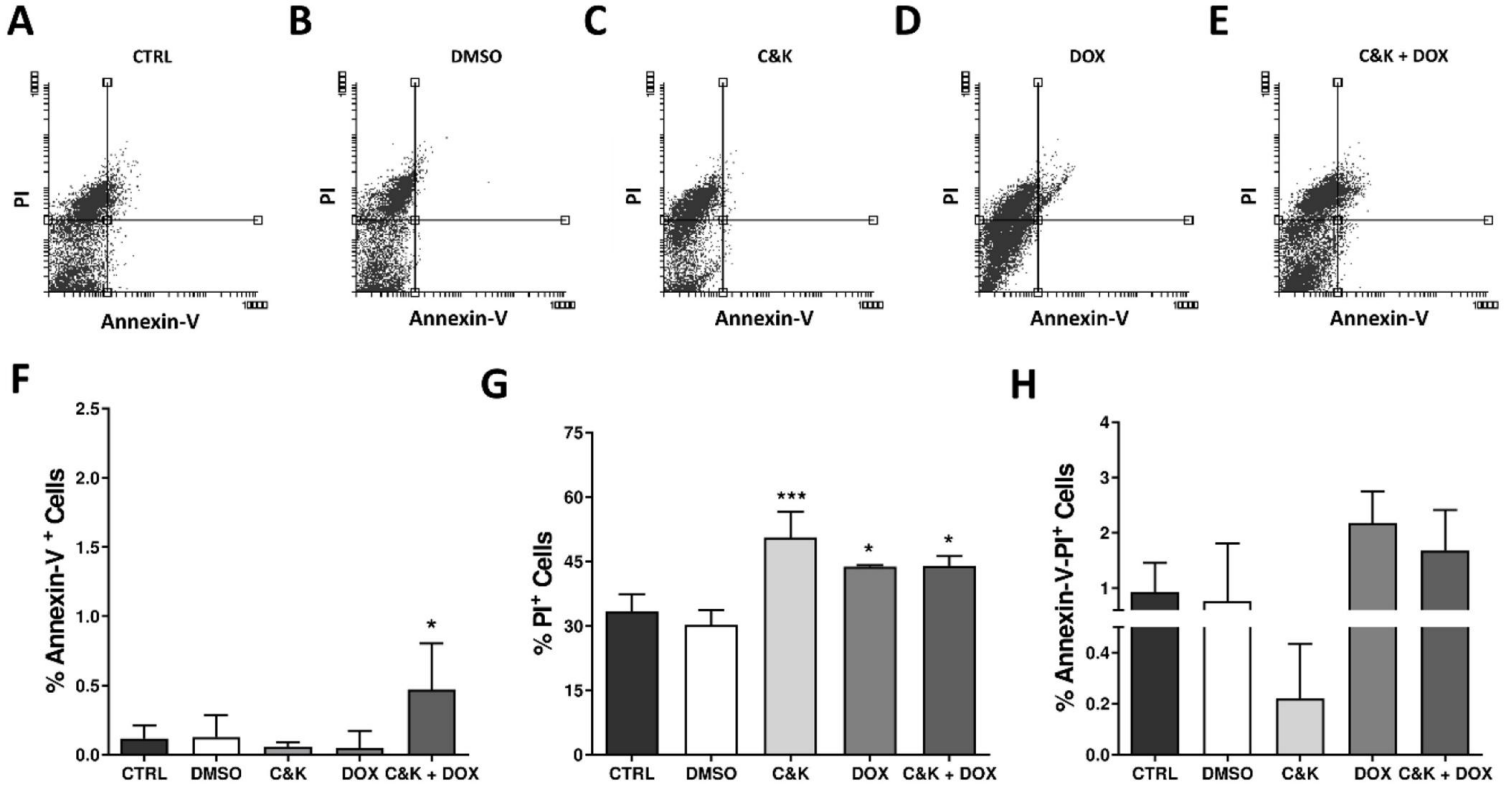
Effect of C&K, doxorubicin, and co‐treatment of C&K with doxorubicin on the type of cell death. MDA‐MB‐231 cells were treated with 1% DMSO, the IC_50_ of C&K, doxorubicin (DOX), or co‐treated with C&K and doxorrubicin for 48 h, stained with Annexin‐V‐FITC and propidium iodide (PI), and evaluated by flow cytometry (A–E). Annexin‐V‐ positive cells (F), PI‐positive cells (G), and Annexin‐V/PI positive cells (H). The data represent the mean ± SEM of three independent experiments. *p < 0.05, **p < 0.001, ***p < 0.0001, in relation to control.

Additionally, MDA‐MB‐231 cells were labelled with a cell cycle staining solution and analyzed by flow cytometry to evaluate the effects of C&K, as well as the combination of C&K with doxorubicin, on cell cycle progression. Cells treated with DMSO showed no alterations in the cell cycle compared to the untreated control (Figure 4A,B,F). Our results demonstrate that treatment with 30.40 μM C&K, 2.50 μM doxorubicin, or a combination of 17.52 μM C&K with 0.15 μM doxorubicin significantly increased the sub‐G0/G1 population by 5.10‐, 7.73‐, and 5.31‐fold, respectively, suggesting apoptosis. Concurrently, the G0/G1 phase was decreased by 1.52‐, 1.59‐, and 2.14‐fold, respectively (Figure 4C–E,F). These findings indicate that C&K, alone or in combination with doxorubicin, induces cell cycle arrest in MDA‐MB‐231 cells. Together, our data indicate that the increase in the percentage of apoptotic or necrotic cells and cell cycle arrest in the sub‐G0/G1 phase, indicative of apoptosis, supports the cytotoxicity promoted by C&K and the association of C&K with doxorubicin.

**Figure 4:**
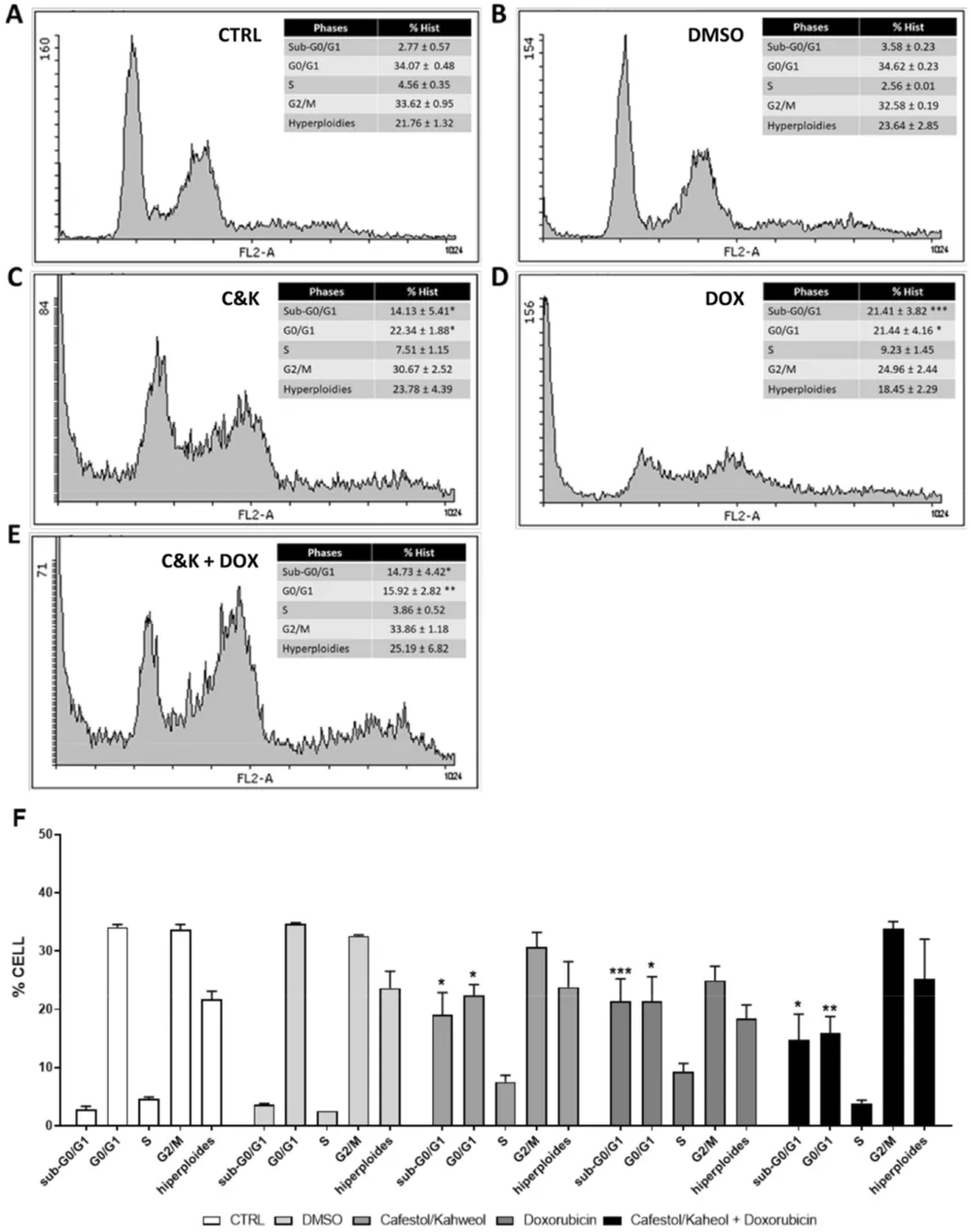
Effect of C&K, doxorubicin, and co‐treatment of C&K with doxorubicin on the cell cycle. MDA‐MB‐231 cells were treated with 1% DMSO, the IC_50_ of C&K, doxorubicin (DOX), or co‐treated with C&K and doxorubicin for 48 h, stained with PI, and evaluated by flow cytometry. (A–F). (A) Control, non‐treated cells. (B) Cells treated with 1% DMSO. (C) Cells treated with IC_50_ of C&K. (D) Cells treated with IC_50_ of DOX. (E) Cells treated with the association of C&K + DOX. Histograms are representative of three independent experiments and show DNA content (*x* axis, PI fluorescence) vs. counts (*y* axis). *p < 0.05, **p < 0.001, ***p < 0.0001, in relation to control.

To interrogate the effect of the association of C&K with doxorubicin on ROS levels, MDA‐MB‐231 cells were labeled with DCFH‐DA and evaluated by flow cytometry. Doxorubicin and the association of C&K with doxorubicin increased 5.09‐ and 1.58‐fold the ROS levels compared to control, respectively (Figure 5).

**Figure 5:**
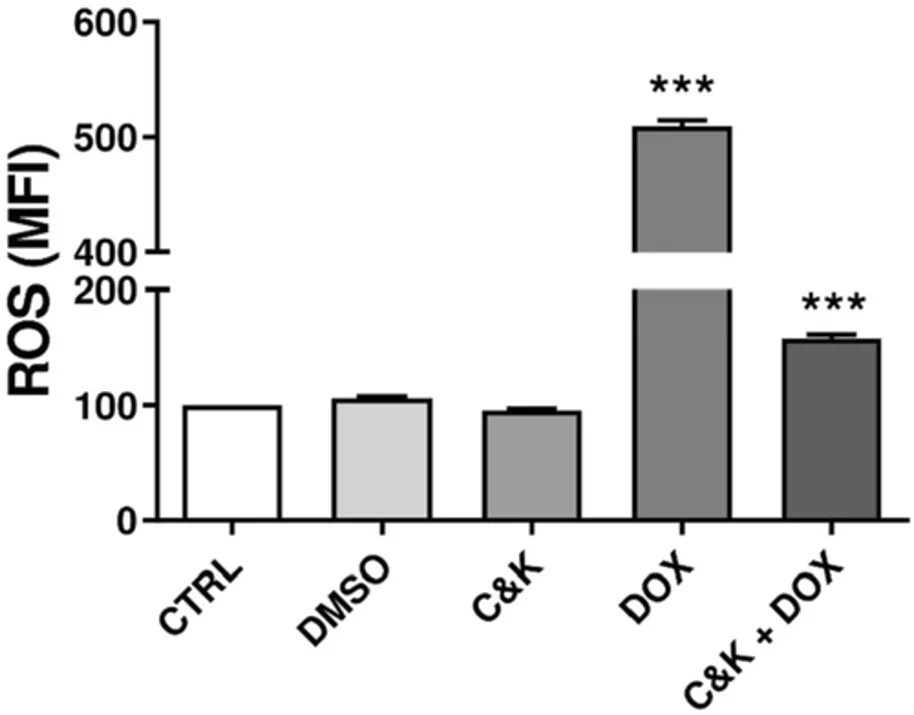
Effect of C&K, doxorubicin, and co‐treatment of C&K with doxorubicin on the ROS levels. MDA‐MB‐231 Cells were treated with 1% DMSO, the IC_50_ of C&K, doxorubicin (DOX), or co‐treated with C&K and doxorubicin for 3 h, stained with 2′,7′‐dichlorofluorescin diacetate (DCFDA), and the ROS levels were measured immediately by flow cytometry. Data are presented as mean ± SEM from three independent experiments. ***p < 0.0001, compared to the control. MFI: Median Fluorescence Intensity.

### 2.4 C&K and the Association of C&K With Doxorubicin Downregulate Pro‐Tumor Pathways in MDA‐MB‐231 Cells

We conducted a microarray assay to determine the Differential Gene Expression (DGE) profile, comparing C&K and control. Our results revealed 762 upregulated and 573 downregulated genes (fold‐change: ±1.5). The upregulated genes were clustered by KEGG (FDR ≤ 0.05) in systemic lupus erythematosus, metabolic pathways, alcoholism, ribosome, Huntington disease, viral carcinogenesis, Parkinson's disease, oxidative phosphorylation, pyrimidine metabolism, and Alzheimer's disease.

The downregulated genes were grouped in the IL‐17 signaling pathway, transcriptional misregulation in cancer, osteoclast differentiation, NF‐kappa B signaling pathway, Th17 cell differentiation, TNF signaling pathway, and NOD‐like receptor signaling pathway (Figure 6A). Moreover, the downregulated genes were grouped in photodynamic therapy‐induced NF‐κB survival signaling by Wikipathway cancer (Figure 6B). Our findings suggest that C&K affects pro‐tumor and inflammation‐related pathways, such as the TNF/NF‐κB pathway, and its cytotoxicity may involve turning off such pathways.

**Figure 6:**
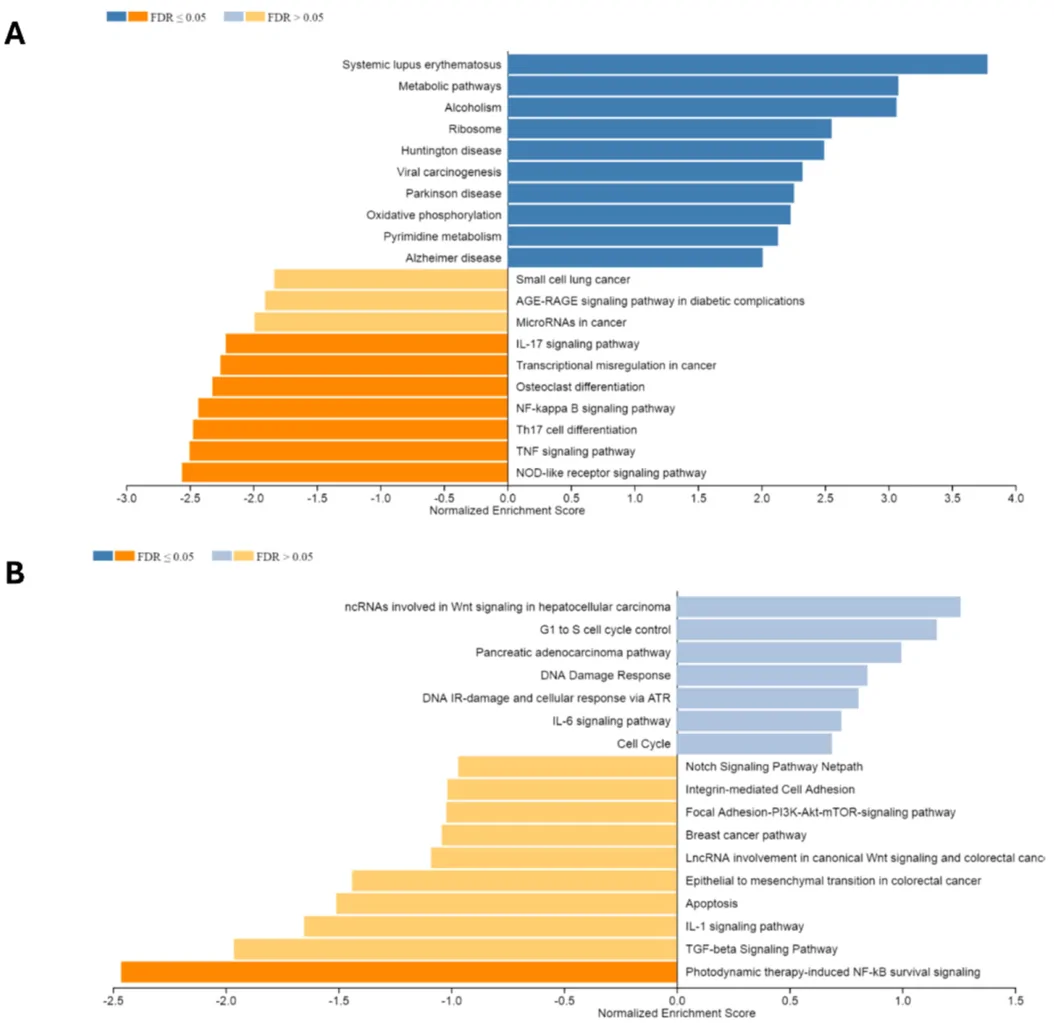
Enrichment of signaling pathways after treatment with C&K using (A) KEGG and (B) Wikipathway cancer databases. The blue bars represent the upregulated signaling pathways, and the orange bars correspond to the downregulated signaling pathways. Bars with a more intense blue or orange color correspond to FDR ≤ 0.05.

We also performed microarray analysis to identify DEGs resulting from the association of C&K with a 10‐fold lower concentration of doxorubicin, which continued to be effective in reducing cell viability (Supporting Information S1: Figure 1), revealing 120 upregulated and 2203 downregulated genes (fold‐change: ±1.5) in cells co‐treated with C&K and doxorubicin when compared to those treated with doxorubicin. The downregulated genes were clustered in RNA transport by KEGG (FDR ≤ 0.05) (Figure 7A) and in DNA mismatch repair, NRF2‐ARE regulation, and Retinoblastoma Gene in Cancer by Wikipathway cancer (Figure 7B). Thus, pathways such as the NRF2‐ARE pathway and retinoblastoma gene in cancer are possible pathways underlying the observed cytotoxic effects.

**Figure 7:**
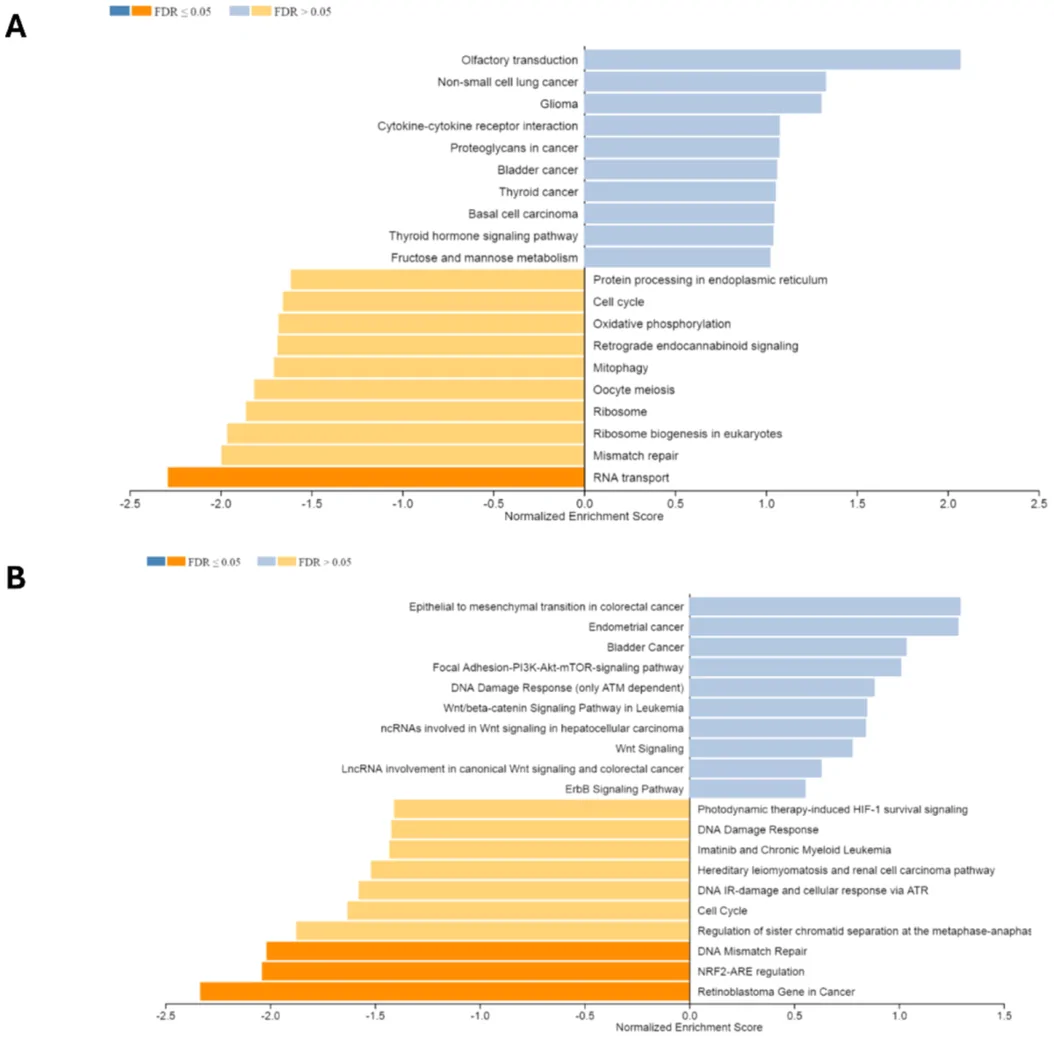
Grouping of DEGs in signaling pathways after co‐treatment of C&K with doxorubicin using (A) KEGG and (B) Wikipathway cancer databases. The blue bars represent the upregulated signaling pathways, and the orange bars correspond to the downregulated signaling pathways. Bars with more intense colors indicate FDR ≤ 0.05.

### 2.5 C&K Decrease NF‐κB Protein Levels

Given that the NF‐κB pathway was downregulated in response to C&K treatment, we sought to validate the microarray results and assess the effect of C&K on NF‐κB protein levels. Treatment with C&K reduced NF‐κB protein levels by 1.39‐fold, whereas doxorubicin and the combination of C&K with doxorubicin increased them by 2.50‐fold and 1.57‐fold, respectively (Figure 8). This result corroborates the data of downregulation of the NF‐κB pathway in response to C&K, reinforcing that the mechanism underlying the effect of C&K involves NF‐κB.

**Figure 8:**
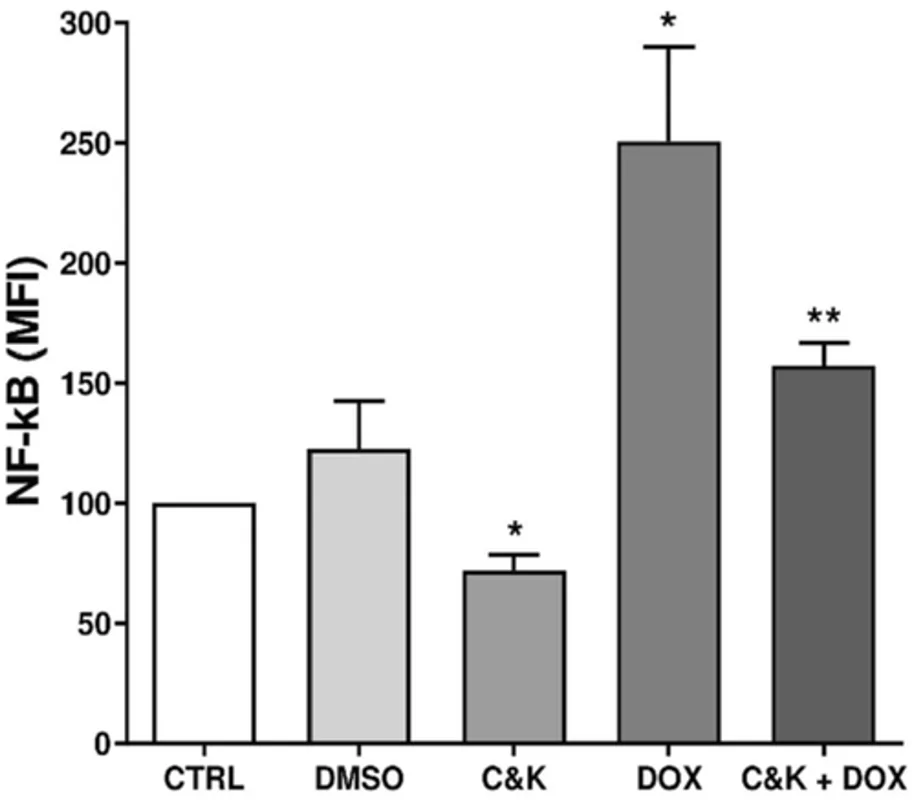
Effect of C&K, doxorubicin, and of the co‐treatment of C&K with doxorubicin ton NF‐κB protein levels. MDA‐MB‐231 cells were treated with 1% DMSO, the IC_50_ of C&K, doxorubicin (DOX), or co‐treated with C&K and doxorubicin for 48 h, fixed, incubated with a primary antibody against NF‐κB, stained with a fluorescent secondary antibody, and evaluated by flow cytometry. Data are presented as mean ± SEM from three independent experiments. *p < 0.05, *p < 0.001, compared to the control. MFI: Median Fluorescence Intensity.

### 2.6 C&K Exhibit Binding Affinity for TNF‐α

As the TNF pathway was downregulated following C&K treatment, we performed a molecular docking simulation to evaluate the interaction between C&K and TNF‐α. The redocking simulation of the complex TNF‐α and its co‐crystallized inhibitor (SPD304) exhibited an RMSD value of 0.3486 Å (Figure 9A). The analysis of the best poses after the docking simulation of the complexes TNF‐α‐C&K showed common aspects, such as Pi‐alkyl interactions with Tyr59′ and Tyr119′ residues (chain B), besides hydrophobic interactions with Leu57, Ty59, Tyr119, Gly121, Gly122 from chain A and Leu57′, Leu120′, Gly121′, Tyr151′ and Ile155′ from chain B, key residues in the active site (Figure 9B,C). In addition, cafestol and kahweol showed a similar binding affinity with the TNF‐α molecular target compared to the co‐crystallized inhibitor SPD304 (Figure 9D,E). The data suggest that the cytotoxic effects of C&K observed may be due to TNF‐α inhibition.

**Figure 9:**
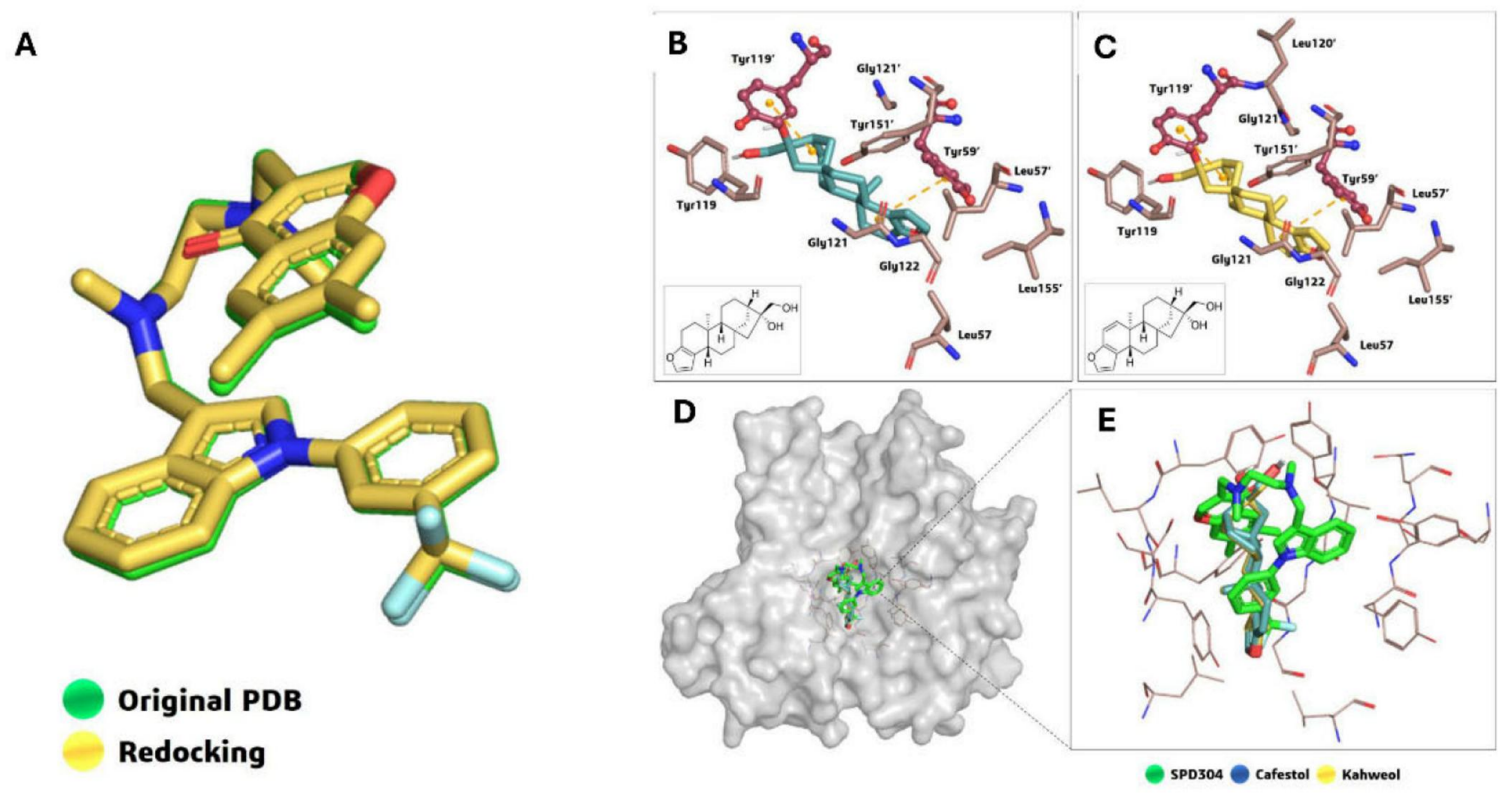
Molecular docking of the interaction of C&K with TNF‐α. Redocking of the best pose result (stick in yellow) of SPD304 co‐crystallized inhibitor (stick in green) at the TNF‐α (PDB ID 2AZ5) active site (A). Interaction diagrams of the best pose results of the TNF‐α‐ligand complex at the active site. Cafestol (blue) (B); Kahweol (yellow) (C). The dashed orange lines represent the Pi‐Alkyl interactions with amino acids (represented by spheres in burgundy color), and residues with hydrophobic interactions are represented by sticks in light burgundy color. Overlap of the pose binding on the TNF‐α active site between ligands Cafestol (blue), Kahweol (yellow), and SPD304 inhibitor (green). Residues of the active site are represented by lines in light burgundy color (D and E).

### 2.7 C&K Gene Signature Is Negatively Related to TNF/NF‐κB Signature in Basal Subtype Breast Cancer Samples

To complement the in vitro assays and evaluate the clinical association between the C&K gene signature (derived from the transcriptome in this study), and the TNF/NF‐κB signature in basal‐like subtype breast cancer samples, we performed a correlation analysis using RNA‐seq data from TCGA. Because our in vitro experiments used the MDA‐MB‐231 triple‐negative breast cancer cell line, we conducted the bioinformatic analysis using the basal‐like subtype cohort, which is available in silico and closely related to TNBC. A statistically significant negative correlation (*r* = −0.5028; *p* = 4.946e‐9; *n* = 120) was observed between the tested signatures (Figure 10A), indicating that patients with increased expression of the C&K signature have decreased expression of the TNF/NF‐κB signature, suggesting an inverse association between these signatures and corroborating the in vitro findings. Furthermore, *BLOC1S1*, a C&K signature gene, stood out as a protective factor for basal‐like breast cancer, in which higher expression levels are associated with greater patient survival (Figure 10B), indicating its prognostic potential, which needs to be further explored and validated in other cohorts.

**Figure 10:**
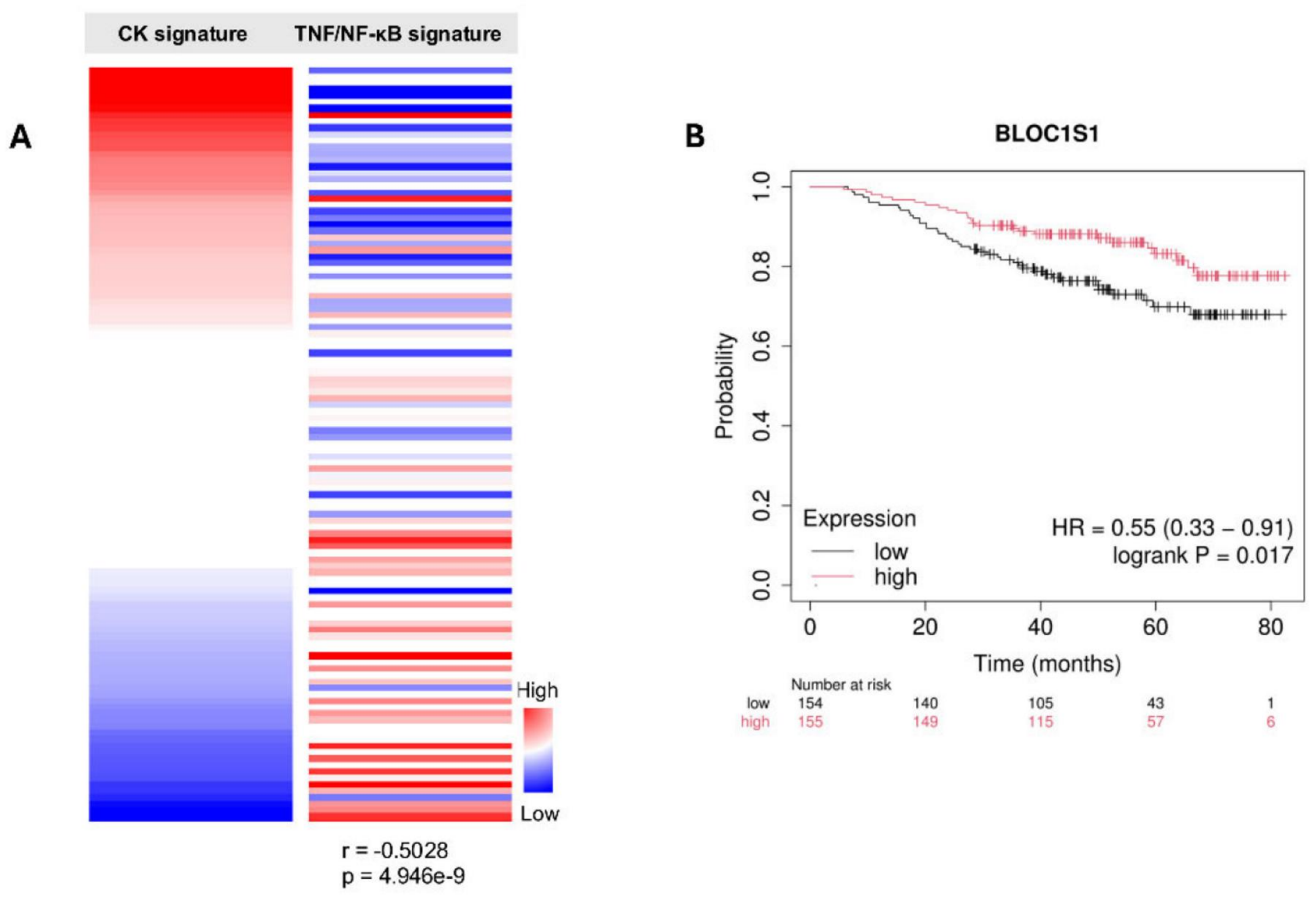
Association analysis, represented by a heatmap and Pearson's rho, between C&K and TNF/NF‐κB signature expression in basal breast cancer cohort of TCGA‐BRCA (A). Kaplan–Meier plot of *BLOC1S1* gene expression (B). *r* = Pearson's rho, *p* = *p*‐value and *n* = 120.

## 3 Discussion

Doxorubicin is a widely used chemotherapy drug for breast cancer treatment. However, the adverse effects limit its use. Furthermore, treatment resistance is another challenge to overcome [3]. In this context, the combination of chemotherapy drugs with bioactives found in natural products has demonstrated anticancer potential, allowing for reduced chemotherapy doses and, consequently, fewer adverse effects [4, 5]. Previously, our group reported that C&K, obtained from the lipid fraction of coffee, maintains the chemical characteristics and an excellent purity level for biological applications [15]. Although the anticancer potential of cafestol and kahweol has been demonstrated individually [9, 16, 17, 18, 19, 20], the effect of the C&K mixture and the combination with chemotherapy drugs on breast cancer needs to be evidenced. Thus, our goal was to evaluate the anticancer effect of C&K alone and in combination with doxorubicin in breast cancer cells.

Our data show that, although the selectivity index was low, C&K was more cytotoxic to MCF‐7 and MDA‐MB‐231 breast cancer cells than the non‐tumorigenic MCF‐10A breast cells, which may be more favorable for minimizing adverse effects. The combination of C&K with doxorubicin was cytotoxic and demonstrated synergism in the aggressive triple‐negative cell line, MDA‐MB‐231, which has not yet been demonstrated in the literature. The relevance of this finding relies on the aggressiveness and lack of therapeutic targets of the triple‐negative subtype. Thus, it requires a broader therapeutic strategy, including combinations of drugs with a role in cell cycle arrest. However, it is less responsive to chemotherapy, resulting in most cases of recurrence. Therefore, more efficient treatments are needed to overcome the low efficacy of current therapy against the triple‐negative subtype [21].

In the literature, studies show that cafestol and kahweol individually increases the cytotoxic effect of chemotherapy drugs. Lima et al. (2017) demonstrated that cafestol increases the cytotoxic effect of cytarabine (Ara‐C), a DNA alkylating drug used in leukemia treatment, in HL60 leukemia cell cultures [19]. In addition, Min et al. (2017) reported synergic effects of kahweol and sorafenib, which were more cytotoxic on A549 lung carcinoma and MDA‐MB‐361 breast cancer cells than a treatment with sorafenib alone [22].

C&K harbor antioxidant properties, including protection against the ROS stimulus driven by chemical agents such as doxorubicin. In our microarray data, we observed a decrease in signaling pathways directly related to the inflammatory response that drives a shift toward oxidative metabolism, including NOD, NF‐κB, and TNF signaling. C&K impaired the ROS increase induced by doxorubicin treatment, suggesting ROS modulation via NF‐κB‐related pathways. This crosstalk between NF‐κB and oxidative stress/ROS signaling has already been explored [23]. Esparza et al. (2013) showed that doxorubicin increases NF‐κB in breast cancer cells [24]. Cafestol and Kahweol act by preventing NF‐κB activation at several steps of the NF‐κB pathway [8]. Regarding NF‐κB, C&K diminished NF‐κB protein levels in cells co‐treated with C&K and doxorubicin compared with doxorubicin alone, suggesting that the doxorubicin‐driven NF‐κB stimulus is attenuated by the antioxidant properties of C&K.

The cytotoxicity promoted by C&K and the combination of C&K with doxorubicin was supported by an increase in the percentage of necrotic cells or apoptotic cells, respectively, and by cell cycle arrest in the sub‐G0/G1 phase, indicative of apoptosis. Molecularly, the downregulation of TNF‐a/NF‐κB signaling pathways, confirmed by the decrease of the NF‐κB protein levels, was observed in response to C&K treatment.

In this context, molecular docking studies were conducted to assess the interaction between C&K and TNF‐α. The docking results were validated by redocking, showing an RMSD value of 0.3684 Å. The active site of TNF‐α is located at the dimer interface and consists of sixteen amino acid residues [25]. C&K interacted similarly in the catalytic site pocket with an RMSD value between poses of 0.134 Å. Of sixteen residues of the active site, twelve showed interactions, including Pi‐Alkyl interactions with two tyrosine residues (Tyr59 and Tyr119), which are considered to have a key role in the accommodation of inhibitors. Interestingly, Tyr119 is considered the most important residue due to the rotation of its angles, which allows accommodation of ligands at the active site and inhibition of TNF‐α [25]. Furthermore, C&K showed a similar binding affinity with the molecular target compared to the co‐crystallized inhibitor SPD304, suggesting that these compounds could bind to the active site of TNF‐α and inhibit this target, which could explain the cytotoxic activity of C&K.

The TNF/NF‐κB pathway is associated with inflammation and pro‐tumoral effects [26, 27]. The C&K anti‐inflammatory effects were observed in human umbilical vein endothelial cells (HUVEC) by decreasing PTGS2 and MCP‐1 genes [7], as well as in prostate cancer cells by inhibition of CCR2, CCR5, and STAT3 [28]. Several studies indicated that coffee consumption has systemic anti‐inflammatory effects [29, 30]. It was demonstrated that a coffee extract reduces *TNFA* and *IL‐6* mRNA along with NF‐κB inhibition in mice [31]. Also, kahweol diminishes liver inflammation in primary Kupffer cells and primary hepatocyte cultures [32]. Our findings suggest an effect of C&K in pro‐tumor and inflammatory‐related pathways, and its cytotoxicity may involve turning off such pathways.

Concerning the downregulated pathways after cotreatment with C&K and doxorubicin, the NRF2‐ARE antioxidant response pathway [33] and retinoblastoma gene in cancer are possible pathways underlying the observed antitumor effects of this treatment. Since doxorubicin acts by generating free radicals [3], the downregulation of the NRF2 signaling pathway, the main defense mechanism against oxidative stress, may be making the tumor cell more sensitive to doxorubicin.

Additional analysis using the TCGA database of basal‐like breast cancer samples, demonstrated a moderate negative correlation between the expression of the C&K signature, composed of the top 12 altered genes, and the TNF/NF‐κB signature, indicating an inverse association between these signatures and corroborating the in vitro analysis. Furthermore, *BLOC1S1*, a C&K‐regulated gene, stood out as a protective factor in basal‐like breast cancer, and further studies are needed in other cohorts to validate this finding.

## 4 Conclusions

C&K increases the cytotoxic effects of doxorubicin and downregulate TNF‐α/NF‐κB pathway in breast cancer cells, highlighting its anticancer potential for further studies.

## 5 Experimental

### 5.1 Compounds and Treatment Conditions

C&K [34] were obtained from green Arabica coffee beans (300*g*) at a ratio of 54.1:45.6 (cafestol:kahweol) and purity exceeding 99%, following procedure detailed elsewhere [34]. In brief, it was used 0.8 L of 2.5 M KOH (Vetec, RJ, Brazil) in methanol (Tedia Company, Fairfield, OH, USA) to direct hot saponification. The mixture was kept in a 3 L flat‐bottomed flask stirred at 800 rpm for 1 h at room temperature (overhead stirrer IKA Eurostar 60 digital package, Staufen, Germany). Adding 0.8 L of water afterward allowed recovery of the C&K mixture into equal volume (0.8 L) of Tert‐Butyl‐Methyl‐Ether (TBME). The resulting organic phase was evaporated, solubilized in 0.3 L of heptane, and partitioned with 0.3 L of aqueous methanol. Once concentreated, the methanolic fraction was concentrated, dissolved in a minimal amount of ethyl acetate/hexane (1:1, v/v), and load onto a chromatography column (CC, 40 × 3 cm id) filled with 30 g of silica gel flash F60 (40–63 µm, Silicycle, Quebec, Canada). The ethyl acetate/hexane (1:1, v/v) also served as the mobile phase during CC purification (ran at 2.5 cm/min). C&K eluted between 14 and 23 fractions collected into 10 mL vials, which were characterized by gas chromatography coupled to quadrupole mass spectrometer (GC‐qMS) confirmed with reference standards. Finally, the solvent mixture was removed by vacuum to constant mass, and the diterpenes were referred to here as a standard mixture. C&K stock solution (10 mg/mL) was prepared in DMSO and stored in aliquots at −20°C. DMSO was used as a control in all assays. C&K (10, 25, 50, 75, and 100 μM) and doxorubicin (D1515, Sigma‐Aldrich, St. Louis, MO, USA) were applied alone for 48 and 72 h and in combination for 48 h.

### 5.2 Cell Culture

MDA‐MB‐231 (ATCC, HTB‐26) and MCF‐7 (HTB22, ATCC) breast cancer cell lines were cultured in RPMI1640 medium (Sigma‐Aldrich, St. Louis, MO, USA) supplemented with 10% FBS, 100 mg/mL penicillin G, 100 U/mL streptomycin, and 2 mM l‐glutamine (Invitrogen, Carlsbad, CA, USA). The MCF‐10A non‐tumorigenic cell line was cultured in DMEM/F12 medium supplemented with 10% FBS, 100 mg/mL penicillin G, 100 U/mL streptomycin, 2 mM l‐glutamine (Invitrogen, Carlsbad, CA, USA), 1 mg/mL EGF, 4 mg/mL insulin and 10 mg/mL hydrocortisone (Sigma‐Aldrich, St. Louis, MO, USA). The cells were cultured in a humidified incubator at 37°C and 5% CO_2_.

### 5.3 MTT Assay

MTT colorimetric assay (Sigma‐Aldrich, St. Louis, MO, USA) was used to evaluate the cytotoxic effect of the treatments. The absorbance was measured at 595, with 630 nm as the reference wavelength, using the LMR‐96i Loccus ELISA reader. The relative number of viable cells was expressed as a percentage of the untreated cells and the IC_50_ values were obtained from dose–response curves, calculated using GraphPad Software 8.0 (GraphPad Inc., San Diego, CA, USA) [35]. The selectivity index (SI) was calculated as the ratio of the MCF‐10A non‐tumorigenic cells' IC_50_ to the IC_50_ of the MCF‐7 or MDA‐MB‐231 breast cancer cell lines, as described by Passos et al. 2015 with modification [13].

### 5.4 Isobologram Construction

The fractional inhibitory concentration index (FIC) of C&K was calculated using the formula IC50 of C&K in association with IC_50_ of C&K alone (IC_50_, 50% inhibitory concentration) in MDA‐MB‐231 cells. The same formula was applied to doxorubicin. The mean FIC was calculated for each combination and then compared to the reference values and reported as very strong synergism (< 0.1), strong synergism (0.1–0.3), synergism (0.3–0.7), moderate synergism (0.7–0.85), slight synergism (0.85–0.90), nearly additive (0.90–1.10), slight antagonism (1.10–1.20), moderate antagonism (1.20–1.45), antagonism (1.45–3.3), strong antagonism (3.3–10) and very strong antagonism (> 10). The synergistic effect between C&K + doxorubicin was analyzed using FIC values to plot the isobologram, according to the method of Chou (2006) [36]. Antagonistic combinations were defined as points above the line of additivity, and synergistic combinations were defined as points below the line.

### 5.5 Detection of Reactive Oxygen Species (ROS)

ROS levels were analyzed in the MDA‐MB‐231 cells treated with C&K and the association of C&K with doxorubicin for 3 h. The cells were then stained with 10 μM 2′‐7′‐dichlorodihydrofluorescein diacetate (DCFH‐DA, Sigma‐Aldrich) for 30 min in the dark, and ROS levels were measured immediately at 500/526 nm excitation/emission wavelengths using a BD FACSCalibur flow cytometer (Becton Dickinson, Franklin Lakes, NJ, USA) and analyzed using BD CellQuest Pro software (Becton Dickinson) [35].

### 5.6 Cell Cycle Analysis

MDA‐MB‐231 cells were treated with C&K and the combination of C&K with doxorubicin for 48 h. After treatment, the cells (5 × 10^5^) were washed with PBS and fixed in 70% (v/v) ice‐cold methanol/PBS for at least 1 h at 4°C. The fixed cells were washed once with PBS and then incubated in PBS supplemented with 10 μg/mL propidium iodide (PI) and 20 μg/mL RNAse at 37°C for 30 min in the dark. For each sample, 10,000 events were acquired using the BD FACSCalibur (Becton Dickinson) flow cytometer and analyzed in BD CellQuest Pro software [37].

### 5.7 Apoptosis/Necrosis Assay

Double staining for annexin V‐fluorescein isothiocyanate (FITC) and PI was performed with the Annexin‐V apoptosis detection kit (Molecular Probes, Eugene, OR, USA). MDA‐MB‐231 cells were treated with C&K and the association of C&K with doxorubicin for 48 h. Cells were then washed twice in cold annexin V‐buffer and centrifuged at 2000 rpm for 5 min. Pellets were resuspended in 20 μL of annexin V FITC, and after 15 min of incubation in the dark, 480 μL of annexin V‐buffer containing 0.5 mg/mL PI was added according to the manufacturer's instructions. Annexin V‐FITC labeling was acquired on a BD FACSCalibur flowcytometer(Becton Dickinson) and analyzed using BD CellQuest Pro software [37].

### 5.8 Flow‐Cytometric Measurement of NF‐kB Protein

The cells were treated with C&K and the association of C&K with doxorubicin for 48 h. Next, the cells were fixed with 4% paraformaldehyde, 0.3% bovine serum albumin (BSA), and 0.5% Triton X‐100 for 20 min and permeabilized with Triton X‐100 (0.5% in PBS) for 25 min. The cells were then washed in PBS three times and incubated with primary antibody for NF‐κB (1:500) (Sc‐8008, Santa Cruz, TX, USA) in the dark for approximately 1 h. Next, cells were washed in PBS three times and incubated with the fluorescence secondary antibody (Alexa 647, ThermoFisher, Waltham, MA, USA) in the dark for approximately 1 h. The cells were washed and the fluorescence collected by flow cytometry on a BD FACSCalibur platform (Becton Dickinson) and analyzed using BD CellQuest Pro software [35].

### 5.9 Microarray Assay and Pathway Analysis

RNA was extracted from cell culture using the RNeasy Mini Kit (Qiagen, Inc.), and RNA purity was determined using an RNA Nano Chip Agilent 2100 Bioanalyzer (Agilent Technology, Inc.). This assay was conducted as previously described by Amorim et al. 2021 [38]. The thresholds for a differentially expressed gene were *p*‐value < 0.05, fold‐change cut off ≥ ±1.5 and a linear step‐up false discovery ratio of *p* ≤ 0.05. Pathway enrichment analysis comparing C&K‐treated vs. untreated cells, and C&K and doxorubicin‐treated vs. doxorubicin‐treated cells, was performed using the online tool WebGestalt (WEB‐based Gene SeT AnaLysis Toolkit) [39, 40], employing the Gene Set Enrichment Analysis (GSEA) method and the KEGG (Kyoto Encyclopedia of Genes and Genomes) [41], and Wikipathway cancer databases [38, 42].

### 5.10 Molecular Docking

The crystallographic structure of the Human Tumor Necrosis Factor alpha (TNF‐α) co‐crystallized with a small inhibitor SPD304 [43] was obtained from the Protein Data Bank (PDB ID: 2AZ5, resolution: 2.10 Å). The active site was centered at the following coordinates: *x*: ‐19.1629, *y*: 73.2356, *z*: 37.2471, within 12 Å. The 3D structures of C&K were constructed in ChemDraw, and then the geometry optimization was performed using the MM2 force field implemented with ChemBio3D v.12.0 (PerkinElmer Informatics, MA, USA) [44]. Molecular docking was performed using the GOLD program (Genetic Optimization for Ligand Docking, Cambridge Crystallographic Data Centre, version 2020.1). The scoring function employed was GoldScore. Polar hydrogen atoms were added to the protein based on the ionization inferred by the program, the number of genetic operations in each run, and other parameters were set as default. Water was excluded from the structure. In addition, the ligands were subjected to 50 iterative runs. Among the 50 runs for each ligand, the pose with the best fitness score was considered and selected for interaction analysis. The analysis of intermolecular interactions and figure building were built in the Pymol program (version 3.8) [45]. Finally, the protocol validation was made by redocking considering RMSD < 2.00 Å. RMSD values between pose solutions were calculated using Discovery Studio Visualizer (Dassault Systèmes BIOVIA, Discovery Studio Modeling Environment, 2017 version, San Diego: Dassault Systèmes, 2016).

### 5.11 The Cancer Genome Atlas (TCGA) Data Analysis

Public data from the TCGA‐BRCA basal breast cancer cohort (*n* = 120) were used for in silico gene expression assessments. The analyses were performed on the UCSC Xena platform [46], from which the RNA‐seq data (log2(expected_count‐deseq. 2 + 1)) were obtained. Pearson's correlation analysis was performed between the mRNA levels of C&K signature and the TNF/NF‐κB [47] gene signature to clinically evaluate the relationship between them [48]. The C&K gene signature is composed of the most altered genes (cutoff > 5.0 or < −5.0) obtained from microarray analysis (= + *BLOC1S1* + *HIST1H4A* + *HIST1H2BB* + *NDUFB3* + *ANKRD36B* ‐ *MALAT1* – *ARL17B* ‐ *BIRC3* ‐ *NFATC2* ‐ *FOSB* ‐ *EGR1*). KMplotter was used to construct a Kaplan‐Meier curve associated with *BLOC1S1* gene expression [49, 50].

### 5.12 Statistical Analysis

Data normality was assessed using the Kolmogorov–Smirnov test. The Mann–Whitney test or Student's *t*‐test was employed to compare two groups, and the Kruskal–Wallis test or one‐way ANOVA was used for more than two groups. Statistical analysis was performed using GraphPad Prism v.5 (GraphPad Inc., San Diego, CA, USA). *p*‐values < 0.05 (two‐sided) were considered statistically significant (* = *p* < 0.05; ** = *p* < 0.01; *** = *p* < 0.001), and post hoc analyses (Tukey's honest significance or Dunn's multiple comparison tests) were applied when necessary.

## Conflicts of Interest

The authors declare no conflicts of interest.

## Supporting information


Supporting File 1

Supporting File 2

Supporting File 3

## Data Availability

The data that support the findings of this study are available on request from the corresponding author. The data are not publicly available due to privacy or ethical restrictions.
