# Supplementary material for: Synergistic Cytotoxicity of Cafestol and Kahweol With Doxorubicin in Breast Cancer Cells Involves TNF‑α Targeting and NF‑κB Downregulation
Source: Arch Pharm (Weinheim). 2026 Oct 7;359(10):e70359. doi: 10.1002/ardp.70359 (PMC13646870; doi:10.1002/ardp.70359)
Supplement: Supplementary file 1 [file ARDP-359-e70359-s002.docx]

**Supplementary table 1.** Cytotoxicity results for cafestol and kahweol (C&K) and doxorubicin (DOX) in MCF-7 and MDA-MB-231 breast cancer cells and MCF-10A non-tumor breast cell.

| **Hours** | **Treatment** | **IC_50_ MCF-7** | **IC_50_ MDA-MB-231** | **IC_50_ MCF-10A** | ***SI MCF-10A/MCF-7** | ***SI MCF-10A/MDA-MB-231** |
| --- | --- | --- | --- | --- | --- | --- |
| 48 | C&K | 22.14 ± 4.10 | 30.37 ± 3.80 | 33.10 ± 0.21 | 1.50 | 1.09 |
|  | DOX | 6.71 ± 1.00 | 2.50 ± 2.70 | 18.90 ± 1.54 | 2.82 | 1.13 |
| 72 | C&K | 18.12 ± 0.67 | 26.43 ± 1.62 | 30.33 ± 1.42 | 1.67 | 1.15 |
|  | DOX | 2.68 ± 0.51 | 1.48 ± 0.07 | 3.24 ± 1.07 | 1.21 | 2.19 |

*SI = Selective Index.

**Supplementary table 2.** Fractional inhibitory concentration of cafestol and kahweol (C&K) with doxorubicin (DOX) in MDA-MB-231 breast cancer cells.

| **Drug combinations (µM)** | **Fractional Inhibitory Concentration (FIC)** | **Description** |
| --- | --- | --- |
| 17.52 C&K + 0.15 DOX | 0.63 | Synergism |
| 13.24 C&K + 0.31 DOX | 0.55 | Synergism |
| 9.98 C&K + 0.62 DOX | 0.56 | Synergism |
| 4.89 C&K + 1.25 DOX | 0.66 | Synergism |
