# Supplementary material for: Synergistic Cytotoxicity of Cafestol and Kahweol With Doxorubicin in Breast Cancer Cells Involves TNF‑α Targeting and NF‑κB Downregulation
Source: Arch Pharm (Weinheim). 2026 Oct 7;359(10):e70359. doi: 10.1002/ardp.70359 (PMC13646870; doi:10.1002/ardp.70359)
Supplement: Supplementary file 2 [file ARDP-359-e70359-s003.docx]

**Title: Synergistic cytotoxicity of cafestol and kahweol with doxorubicin in breast cancer cells involves TNF‑α targeting and NF‑κB downregulation**

Layane D. Souza ^1#^, Fábio Junior M. Novaes ^3,7#^, Carlos Luan A. Passos ^2#^, Ísis S. S. de Amorim ^1,2#^, Pedro Nicolau-Neto ^4^, Juliana A. Rodrigues ^1^, Adilson F. Teixeira ^1^, Christian Ferreira ^2^, Nathalia Alexia N. dos Santos ^2^, Priscila G. Camargo ^5^, Marcelle L. F. Bispo ^5^, Camilo Henrique S. Lima ^6^, Bruno Ricardo B. Pires ^1^, Adenilson S. da Fonseca ^1^, Claudia M. Rezende ^7^, Eliane Fialho ^2*^, Andre Luiz Mencalha ^1*^

^#^ These authors contributed equally to this work.

^1^ Department of Biophysics and Biometrics, Roberto Alcantara Biology Institute, State University of Rio de Janeiro, Rio de Janeiro, 20331-050, Brazil.

^2^ Functional Foods Laboratory, Nutrition Institute, Federal University of Rio de Janeiro, Rio de Janeiro, 21941-590, Brazil.

^3^ Chemistry Department, Viçosa Federal University, Viçosa, 36570-900, Brazil.

^4^ Molecular Carcinogenesis Program, National Cancer Institute, Rio de Janeiro, 20230-130, Brazil.

^5^ Laboratory of Synthesis of Medicinal Molecules, Chemistry Department, State University of Londrina, Londrina, 86057-970, Brazil.

^6^ Chemistry Institute, Federal University of Rio de Janeiro, Rio de Janeiro, 21941-909, Brazil.

^7^ Aromas Analysis Laboratory, Chemical Institute, Federal University of Rio de Janeiro, Rio de Janeiro, 21941-895, Brazil.

*Corresponding author:

Prof. Andre Luiz Mencalha, PhD. Departamento de Biofísica e Biometria, Instituto de Biologia Roberto Alcântara Gomes, Universidade do Estado do Rio de Janeiro, UERJ, Vila Isabel, Rio de Janeiro, CEP 20551-030, Brazil. E-mail address: [andre.mencalha@uerj.br](mailto:andre.mencalha@uerj.br) or [almencalha@yahoo.com.br](mailto:almencalha@yahoo.com.br). Tel number: +55 21 2334 2058.

Profª. Eliane Fialho de Oliveira, PhD. Departamento de Nutrição Básica e Experimental, Instituto de Nutrição Josué de Castro, Centro de Ciências da Saúde, Universidade Federal do Rio de Janeiro, UFRJ, Caixa Postal 68041, Cidade Universitária, Ilha do Fundão, Rio de Janeiro, CEP 21941-590, Brazil. E-mail address: [fialho@nutricao.ufrj.br](mailto:fialho@nutricao.ufrj.br). Fax number: + 55 21 2280 8343. Tel number: +55 21 3938 6799.

**Supplement Figure 1.** Cell viability in response to C&K and association of C&K with a 10-fold lower concentration of doxorubicin for 48h in MDA-MB-231 cells. CTRL: control; DMSO: dimethyl sulfoxide; C&K: cafestol & kahweol; DOXO: doxorubicin; * p < 0.05.
