# Supplementary material for: Synergistic Cytotoxicity of Cafestol and Kahweol With Doxorubicin in Breast Cancer Cells Involves TNF‑α Targeting and NF‑κB Downregulation
Source: Arch Pharm (Weinheim). 2026 Oct 7;359(10):e70359. doi: 10.1002/ardp.70359 (PMC13646870; doi:10.1002/ardp.70359)
Supplement: Supplementary file 3 [file ARDP-359-e70359-s001.doc]

**Supplemental Material: Novel Compounds and Biological Screening Results**

*(Please replace all information and advice given in blue before submission)!*

Title of Manuscript: **Synergistic cytotoxicity of cafestol and kahweol with doxorubicin in breast cancer cells involves TNF‑α targeting and NF‑κB downregulation**

Authors: Layane D. Souza 1#, Fábio Junior M. Novaes 3,7#, Carlos Luan A. Passos 2#, Ísis S. S. de Amorim 1,2#, Pedro Nicolau-Neto 4, Juliana A. Rodrigues 1 , Adilson F. Teixeira 1, Christian Ferreira 2, Nathalia Alexia N. dos Santos 2, Priscila G. Camargo 5, Marcelle L. F. Bispo 5, Camilo Henrique S. Lima 6, Bruno Ricardo B. Pires 1, Adenilson S. da Fonseca 1, Claudia M. Rezende 7, Eliane Fialho 2*, Andre Luiz Mencalha 1*

Affiliations:

^1^ Department of Biophysics and Biometrics, Roberto Alcantara  Biology  Institute,  State  University  of Rio de Janeiro, Rio de Janeiro, 20331-050, Brazil.

^2^ Functional Foods Laboratory, Nutrition Institute, Federal University of Rio de Janeiro, Rio de Janeiro, 21941-590, Brazil.

^3^ Chemistry Department, Viçosa Federal University, Viçosa, 36570-900, Brazil.

^4^ Molecular Carcinogenesis Program, National Cancer Institute, Rio de Janeiro, 20230-130, Brazil.

^5^ Laboratory of Synthesis of Medicinal Molecules, Chemistry Department, State University of Londrina, Londrina, 86057-970, Brazil.

^6^ Chemistry Institute, Federal University of Rio de Janeiro, Rio de Janeiro, 21941-909, Brazil.

^7^ Aromas Analysis Laboratory, Chemical Institute, Federal University of Rio de Janeiro, Rio de Janeiro, 21941-895, Brazil.

Corresponding author – full address:

Prof. Andre Luiz Mencalha, PhD. Departamento de Biofísica e Biometria, Instituto de Biologia Roberto Alcântara Gomes, Universidade do Estado do Rio de Janeiro, UERJ, Vila Isabel, Rio de Janeiro, CEP 20551-030, Brazil. E-mail address: [andre.mencalha@uerj.br](mailto:andre.mencalha@uerj.br) or almencalha@yahoo.com.br. Tel number: +55 21 2334 2058.

*(All information above can be given in the same way as in the original manuscript. All chemical drawing software packages offer the possibility to create an InChI code from the drawn structure. Please check the corresponding handbook if you have any problems. The concentration dimension should be given within the table).*

| **Compound No.** | **InChI** | **Biological Activity ()^a^** |
| --- | --- | --- |
|  | no new compounds reported |  |
|  |  |  |
|  |  |  |
|  |  |  |
|  |  |  |
|  |  |  |
|  |  |  |
|  |  |  |
|  |  |  |
|  |  |  |
|  |  |  |

^a^ Brief description of screening procedure incl. reference.
